# Total Chemical Synthesis of a Heterodimeric Interchain Bis-Lactam-Linked Peptide: Application to an Analogue of Human Insulin-Like Peptide 3

**DOI:** 10.1155/2013/504260

**Published:** 2013-10-28

**Authors:** John Karas, Fazel Shabanpoor, Mohammed Akhter Hossain, James Gardiner, Frances Separovic, John D. Wade, Denis B. Scanlon

**Affiliations:** ^1^Bio21 Institute, University of Melbourne, Melbourne, VIC 3010, Australia; ^2^The Florey Institute of Neuroscience and Mental Health, University of Melbourne, Melbourne, VIC 3010, Australia; ^3^School of Chemistry, University of Melbourne, Melbourne, VIC 3010, Australia; ^4^CSIRO Materials Science & Engineering, Clayton, VIC 3168, Australia; ^5^MRC Laboratory of Molecular Biology, Cambridge CB2 2QH, UK; ^6^The Florey Department of Neuroscience and Mental Health, University of Melbourne, Melbourne, VIC 3010, Australia; ^7^Department of Chemistry, University of Adelaide, Adelaide, SA 5005, Australia

## Abstract

Nonreducible cystine isosteres represent important peptide design elements in that they can maintain a near-native tertiary conformation of the peptide while simultaneously extending the *in vitro* and *in vivo* half-life of the biomolecule. Examples of these cystine mimics include dicarba, diselenide, thioether, triazole, and lactam bridges. Each has unique physicochemical properties that impact upon the resulting peptide conformation. Each also requires specific conditions for its formation via chemical peptide synthesis protocols. While the preparation of peptides containing two lactam bonds within a peptide is technically possible and reported by others, to date there has been no report of the chemical synthesis of a heterodimeric peptide linked by two lactam bonds. To examine the feasibility of such an assembly, judicious use of a complementary combination of amine and acid protecting groups together with nonfragment-based, total stepwise solid phase peptide synthesis led to the successful preparation of an analogue of the model peptide, insulin-like peptide 3 (INSL3), in which both of the interchain disulfide bonds were replaced with a lactam bond. An analogue containing a single disulfide-substituted interchain lactam bond was also prepared. Both INSL3 analogues retained significant cognate RXFP2 receptor binding affinity.

## 1. Introduction

Cysteine-rich peptides such as conotoxins and insulin-like peptides are an increasingly important class of biomolecules. They usually possess intricately folded, sometimes knotted, structures and some have been developed as treatments for a variety of conditions, such as pain [1, 2], cancer [3], diabetes mellitus [4], and heart failure [5, 6]. As such, much work is being undertaken to optimise their pharmacological properties so that new lead compounds are developed for preclinical evaluation. Disulfide bonds play a critical role in maintaining the peptide conformation and biological activity of these molecules. However, they are susceptible to reduction *in vivo*, as part of the normal degradative process which, in turn, can disrupt the three-dimensional structure and lead to loss of activity. In order to stabilise peptide structures, numerous disulfide bond mimics have been developed. Guo et al. substituted a diselenide for a disulfide bond in a sunflower trypsin inhibitor which retained high potency [7]. Armishaw et al. also applied this further to an *α*-conotoxin, which maintained full biological activity and had enhanced stability under biologically reducing conditions [8]. This same model peptide was also prepared with thioether bonds as cystine mimics, and a similar outcome was achieved with respect to both activity and stability [9]. Further, Holland-Nell and Meldal reported that 1,4-triazoles using the copper(I)-catalysed azide-alkyne cycloaddition can also be a useful cystine isostere [10]. Further work in this area has demonstrated that 1,5-triazoles, produced via ruthenium(II) catalysis, can be an even more effective mimic [11]. Numerous dicarba analogues of cystine-containing peptides have also been prepared [12–15] and shown to possess near-native structure and extended *in vivo* stability. Monosubstituted dicarba bond analogues of the heterodimeric peptides relaxin-3 [16] and insulin-like peptide 3 (INSL3) [17] have also been prepared and evaluated.

The incorporation of lactam bridges in peptides has been widely reported in the literature. Such linkages have been employed as both “staples” in order to stabilise *α*-helices and other secondary structures [18–20] and as a strategy for generating small cyclic libraries [21]. They have also found use of stable cystine isosteres [21–28] as an alternative to the previous methods described. These linkages have two key structural characteristics: (i) there are dual orientations of the asymmetrical amide bond; (ii) bridge length can vary. The direction of the lactam bond can have either a negligible effect on binding and biological activity [22] or a dramatic one. Interestingly, Hargittai and coworkers found that an *α*-conotoxin analogue with a Lys/Glu lactam bond was 4,000 times more potent than the “inverted” Glu/Lys analogue [23]. Different pairings of side-chain carboxylate (Asp, Glu) and amine (Dpr, Dab, Orn, Lys) residues will of course vary the length of the lactam bridge which can also affect peptide conformation and activity [24–26]. The most common residue used is an aspartyl/2,3-diaminopropionyl pairing, since the resultant side-chain to side-chain amide bond will result in the same number of atoms as a cystine bond.

There have been a number of methods developed for synthesising lactam-containing peptides [22, 26–28]. Earlier work focussed on a Boc/Bzl-based strategy, employing the base-labile O-Fm and Fmoc protecting groups for orthogonal protection of carboxylate and amine side-chains, respectively. Typically, the lactam is formed on the solid support, followed by HF cleavage and RP-HPLC. This methodology has been extended to assemble bis-lactam analogues [18] including peptides with overlapping lactam bonds [19]. The latter was performed by employing hydrazine labile O-Dmab and Dde protecting groups in addition to the 9-fluorenylmethyl-based ones as semiorthogonal protection. Thurieau et al. devised a solution-phase approach whereby the N-terminus and a lysine residue were protected until after lactamization in order to yield one discrete product [24]. Allyl/Alloc protection is a common strategy when using an “on-resin” Fmoc/tBu approach [21, 23, 24, 28] as it is the extremely acid-labile and more convenient O-2-PhiPr/Mtt pair [20, 29].

**Figure 1:**
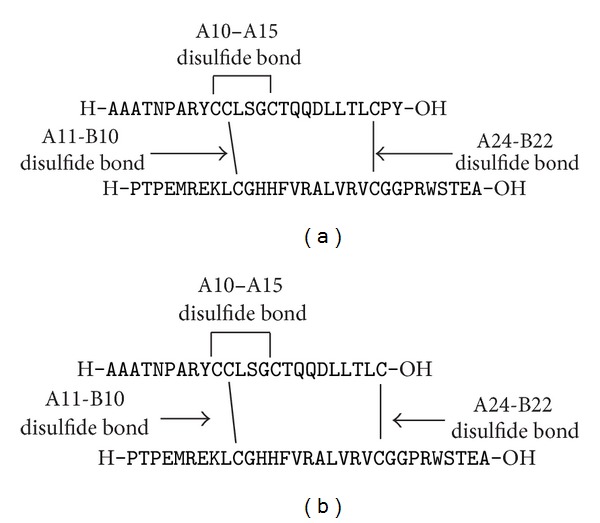
Primary structure of (a) human INSL3 and (b) its analogue ΔA25/26 human INSL3.

INSL3 was chosen as the model system to evaluate lactam bonds as cystine isosteres in complex peptide structures. INSL3 is a hormone which has been shown to play an important role in testicular descent during sexual development [30]. It is heterodimeric in nature with a 26 residue A-chain and a 31 residue B-chain. It contains a disulfide bonding configuration, that is, characteristic of the insulin/relaxin superfamily [31] with one intra-A-chain and two interchain (between A and B) cystine bonds which stabilize the three-dimensional structure of the peptide (Figure 1(a)). Its structure-activity relationship has been studied in detail, and both the A- and B-chains are required for RXFP2 receptor activation [32–34]. Further, an analogue of the INSL3 B-chain alone was shown to be a potent RXFP2 antagonist [35]. Recently, Büllesbach and Schwabe prepared analogues of human INSL3 in which one or the other of the two interchain disulfide bonds were replaced with a lactam bond, in which the purpose was to study the role of the native cystine (A11-B10) and (A24-B22) bonds in both binding and receptor activation [29]. Their synthetic strategy involved native chemical ligation of two A-chain fragments followed by a second ligation with the B-chain. While very effective, the methodology requires the preparation of peptide thioesters as ligation intermediates prior to subsequent off-resin preparation of the mono-intrachain lactam INSL3 analogue. 

Moreover, to date, there have been no reports of a chemical synthesis of a bis-lactam cross-linked heterodimeric peptide. Consequently, in this study, we undertook an examination of the feasibility of a total step-wise synthesis of, first, a monolactam A24-B22 analogue and, subsequently, a bis-lactam A11-B10/A24-B22 analogue of INSL3. Our novel strategy involved the continuous assembly of both the A- and B-chains, plus the lactam bridges on the same solid support, that is, without using preformed peptide fragments. In order to simplify assembly, the C-terminus of the A-chain was truncated at position 24, omitting Pro^A25^ and Tyr^A26^ that results in the analogue ΔA25/26 human INSL3 (Figure 1(b)). Although receptor activation is somewhat diminished for this particular analogue, it still demonstrates significant binding affinity compared to that of the native form [32] and hence was deemed to be a suitable model system to evaluate the synthetic feasibility of assembling these lactam analogues. We describe herein the synthesis of ΔA25/26 human INSL3 having a lactam substitution of Cys^A24,B10^ as well as a first ever reported assembly of a heterodimeric analogue with two interchain lactam bridges, namely, ΔA25/26 human INSL3 bis-lactam A11-B10/A24-B22. The cognate RXFP2 G protein-coupled receptor binding affinity of the analogues was also evaluated.

## 2. Materials and Methods

### 2.1. Solid Phase Peptide Synthesis

All peptides were assembled on a CEM Liberty microwave peptide synthesiser (DKSH, Australia), except for when an unusual amino acid derivative was used whereby it was coupled manually. Standard Fmoc amino acids were obtained from GL Biochem (China) as was HATU and Boc anhydride. Fmoc-L-Asp-OtBu, Fmoc-L-Dpr(ivDde)-OH, Fmoc-L-Dpr(Mmt)-OH, and Fmoc-L-Asp(O-2-PhiPr)-OH were sourced from Novabiochem (Australia). Piperidine, hydrazine, DIPEA, NMP, TIPS, DODT, TFA, iodine, and DOWEX ion exchange resin were obtained from Sigma-Aldrich (Australia). DCM and DMF were purchased from Ajax Finechem P/L (Australia). Fmoc-L-Ala-PEG-PS resin with a substitution of 0.2 mmol/g was obtained from Applied Biosystems (Australia).

### 2.2. RP-HPLC Purification and Analysis

All peptides were analysed on an Agilent 1100 Series HPLC (Australia) with an Agilent Eclipse XDB-C18 column (5 *μ*m, 4.6 × 150 mm). The buffer system used was 0.1% TFA in water (buffer A) and 0.1% TFA in acetonitrile (buffer B). A typical gradient was 0–80% buffer B over 40 minutes at a 1 mL/min flow rate with the detection at 220 nm. Purification was carried out on an Agilent 1200 Series HPLC using either an Agilent Eclipse XDB-C18 column (5 *μ*m, 9.4 mm × 250 mm) or a Phenomenex Synergi Hydro C18 column (4 *μ*m, 21.2 × 50 mm). A typical gradient of 0–60% buffer B over 60 minutes at a flow rate of 5 mL/min was used. 

### 2.3. Mass Spectrometry

The linear and intermediate peptides were characterised on an Agilent 6510 Dual ESI QTOF mass spectrometer (Australia).

### 2.4. Receptor Binding Assay

The receptor binding affinity of both analogues was determined in HEK-293T cells stably transfected with RXFP2 as previously described [32]. Briefly, a single concentration of europium-labelled INSL3 (0.3 nM) [36] was used in the presence of increasing concentration of the unlabelled INSL3 and the analogues (0.01 nM–1 *μ*M). The data was analysed using GraphPad PRISM 4 (GraphPad Inc., San Diego, USA) and expressed as mean ± SEM of three independent experiments. The statistical differences in pK_*i*_ values were calculated using one-way ANOVA coupled to Bonferroni's multiple comparison test for multiple group comparisons.

### 2.5. Synthesis of ΔA25/26 Human INSL3 Monolactam A24-B22

INSL3 B-chain was assembled on Fmoc-L-Ala-PEG-PS resin via microwave-assisted SPPS on a 50 *μ*mol scale using an Fmoc SPPS approach. ivDde-protected diaminopropionic acid (Dpr) was used in place of Cys^B22^, and Cys^B10^ was incorporated with acetamidomethyl (Acm) protection. After chain elongation, the N-terminus of the resin-bound peptide was Boc-protected using di-tert-butyl carbonate in the presence of DIPEA, followed by treatment with a solution of 3% hydrazine (5 × 3 minutes) to cleave the ivDde group. The free amine at the side-chain was then acylated via the HATU activated side-chain of Fmoc-L-Asp-OtBu, and the A-chain was then assembled on the same resin. Cys^A11^ was also incorporated with Acm protection. The peptide was then TFA-cleaved, isolated, and then RP-HPLC-purified. The free sulfhydryls at residues Cys^A10^ and Cys^A15^ were then oxidised with 2 equivalents of iodine in a 50% acetic acid aqueous solution at a concentration of 1 mg/mL for 30 minutes. Excess iodine was quenched with DOWEX ion exchange resin, followed by a second RP-HPLC purification. The Cys^A11^-Cys^B10^ intermolecular disulfide bond was then formed via oxidative cleavage of both Cys(Acm) residues using 20 equivalents of iodine [37]. After a similar workup to the previous oxidation, the crude product was RP-HPLC-purified to a high level and characterised via ESI-MS which gave a molecular mass of 6011 Da (theory: 6011.0). The final mass recovery was 700 *μ*g (yield 1.8% relative to starting crude cleaved peptide). 

### 2.6. Synthesis of ΔA25/26 Human INSL3 Bis-Lactam A11-B10/A24-B22

A similar synthetic strategy was employed for this synthesis on the same 50 *μ*mol scale; however, the lactam bridge mimicking the Cys^A11^-Cys^B10^ bond was also formed on the solid support. Thus B-chain was assembled using Dpr(Mmt) and Dpr(ivDde) at positions B10 and B22, respectively. His(Boc) was used instead of His(Trt) at positions B12 and B13 and was incorporated manually under ambient conditions using HOBt/DIC activation. After N-terminal capping and ivDde cleavage, the A-chain was assembled up to position A11 using an Asp(O-2-PhiPr) residue and leaving the N-terminus Fmoc protected. At positions A17 and A18, unprotected Gln was incorporated (via HOBt/DIC activation) instead of the usual Trt side-chain protected derivative. The resin was then treated with 1% TFA (10 × 3 minutes) to cleave the O-2-PhiPr and Mmt protecting groups from the Asp and Dpr side-chains, respectively. After neutralisation with mild base, the resin was treated with 1.5 equivalents of HATU in the presence of DIPEA to form the A11-B10 interchain mimic. A small-scale pilot cleavage was performed in order to determine the success of this on-resin cyclisation. After reaction, the remainder of the A-chain was assembled and the peptide was isolated and purified via RP-HPLC. Oxidation of the Cys^A10^-Cys^A15^ pair was performed using 2 equivalents of elemental iodine as described above. The product was repurified and lyophilised. 20 *μ*g of material at high purity was recovered (0.08% relative to the isolated crude material), and the peptide was characterised via ESI-MS which showed a molecular mass of 5990 Da (theory: 5989.8).

## 3. Results and Discussion

**Figure 2:**
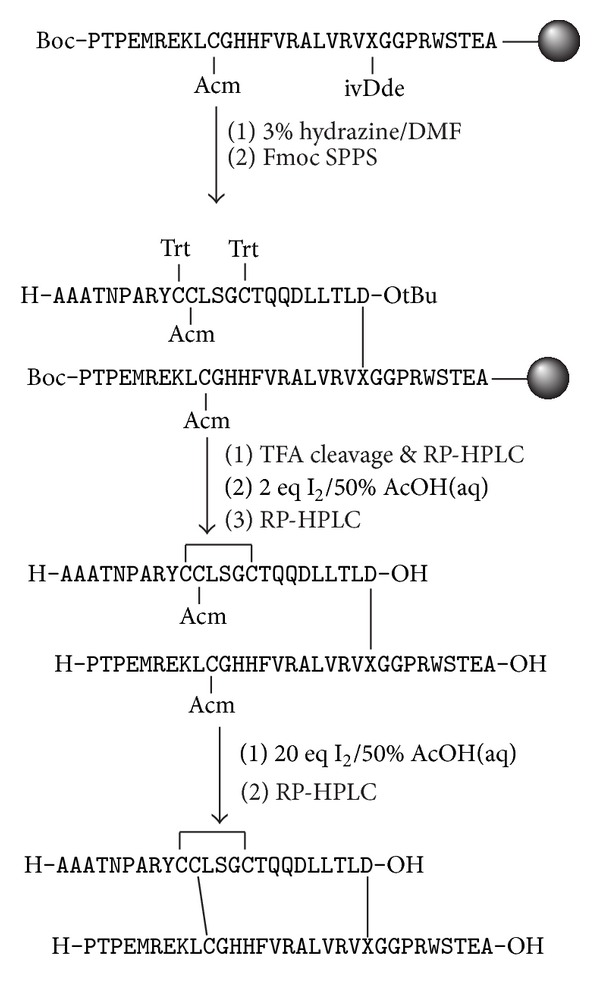
Schematic representation of the synthesis of ΔA25/26 human INSL3 monolactam A24-B22. X = 2,3-diaminopropyl.

**Figure 3:**
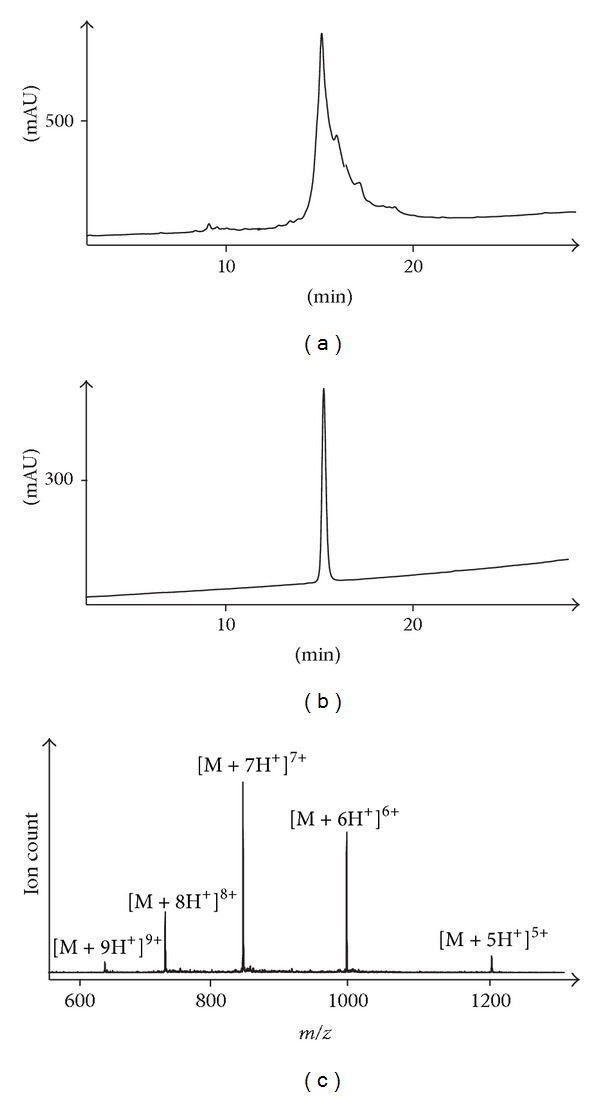
RP-HPLC and ESI-MS data of ΔA25/26 human INSL3 monolactam A24-B22. (a) RP-HPLC of crude ΔA25/26 human INSL3 A24-B22 Cys^A11,B10^ di-Acm species; (b) RP-HPLC of purified ΔA25/26 human INSL3 monolactam A24-B22; (c) ESI-MS of purified ΔA25/26 human INSL3 monolactam A24-B22. HPLC detection wavelength = 220 nm.

An initial assembly of the ΔA25/26 human INSL3 monolactam A24-B22 was undertaken via total chemical synthesis (Figure 2). This analogue was chosen over the ΔA25/26 human INSL3 monolactam A11-B10 analogue solely for reasons of synthetic simplicity with the former having its lactam bond closest to the C-terminus of the synthesis and being theoretically easier to form first before steric crowding became too great a consideration. The microwave-assisted assembly was successful as indicated by RP-HPLC and ESI-MS analysis of the crude S-reduced peptide (Figure 3(a)). The principal impurities were identified to be postcleavage* tert*-butyl adducts which are characteristic of thiol and thioether-containing peptides. After RP-HPLC purification, mild oxidative conditions were employed to form the first A-chain intramolecular (A10-A15) disulfide bond, followed by a second purification. The final oxidation step to form the A11-B10 disulfide bond gave a number of side products which is a common outcome from treating peptides with a large excess of iodine [37]. Trp, Tyr, and Met residues can be modified, all of which are present in INSL3. Furthermore, misfolded isomers and dimers can occur due to disulfide shuffling. Because of this complex mixture, a two-step purification was performed, firstly using a conventional C18 Agilent column, followed by a second C18 Phenomenex column with polar residues bonded to the stationary phase. The rationale behind this strategy was to achieve an alternative selectivity from the chromatography such that most impurities coeluting with the parent compound during the first isolation will be separable during the second pass through the column. This strategy yielded sufficient peptide to be obtained in high purity (Figures 3(b) and 3(c)) which enabled the analogue to be evaluated in the RXFP2 receptor-binding assay. 

**Figure 4:**
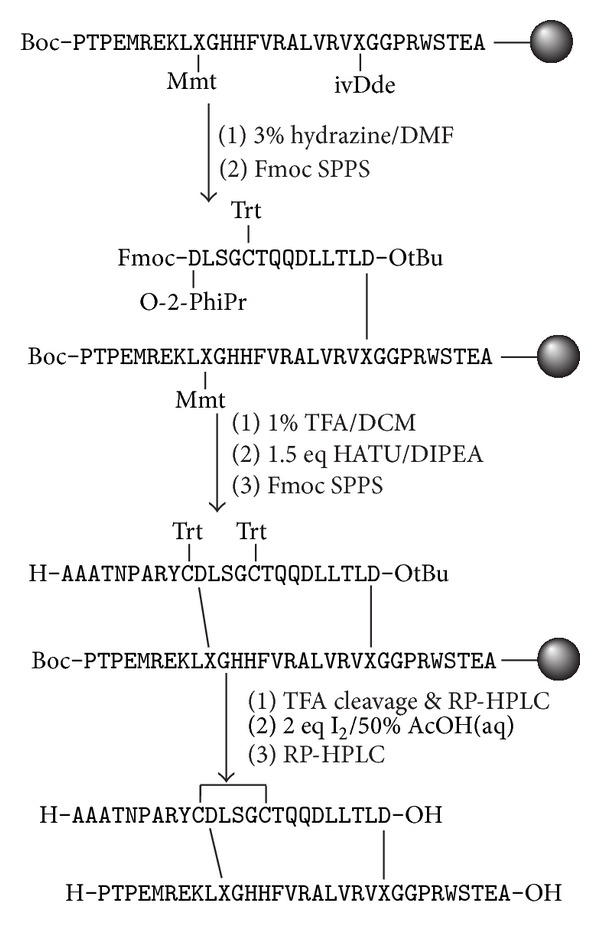
Schematic representation of the synthesis of ΔA25/26 human INSL3 bis-lactam A11-B10/A24-B22. X = 2,3-diaminopropyl.

**Figure 5:**
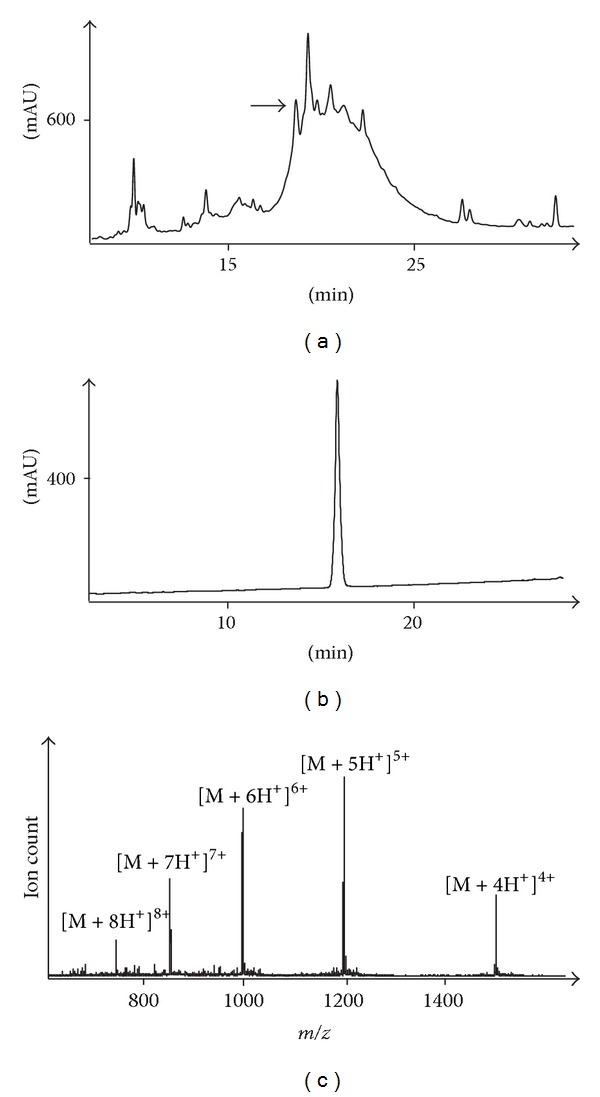
RP-HPLC and ESI-MS data of ΔA25/26 human INSL3 bis-lactam A11-B10/A24-B22. (a) RP-HPLC of crude Fmoc-AspA^10^ ΔA25/26 INSL3 A11-B10/A24-B22; (b) RP-HPLC of purified ΔA25/26 human INSL3 bis-lactam A11-B10/A24-B22; (c) ESI-MS of purified ΔA25/26 human INSL3 bis-lactam A11-B10/A24-B22. HPLC detection wavelength = 220 nm.

Assembly of the ΔA25/26 human INSL3 bis-lactam A11-B10/A24-B22 analogue B-chain (Figure 4) also proceeded smoothly as verified by a small-scale cleavage of the peptide resin. After ivDde deprotection at Dpr^B22^, the A-chain was assembled up to Fmoc-Asp^A11^. RP-HPLC analysis indicated that the target intermediate was the major product. It was thought that forming the intermolecular lactam bond at this point was preferable, since extending the A-chain beforehand would increase the steric crowding on the resin, making it difficult to drive the lactamization to completion. Using the same rationale, Boc-protected His^B11^ and His^B12^ and unprotected Gln^A17^ and Gln^A18^ were also used, instead of the bulkier trityl-based derivatives. Deprotection of Asp^A11^ and Dpr^B10^ with dilute TFA was monitored colorimetrically via Trt cleavage; however, it was not possible to determine whether full removal of the O-2-PhiPr moiety occurred. A trial TFA deprotection showed that this protecting group would cleave off regardless. After neutralisation of the resin, the amide bond between residues A11 and B10 was formed. RP-HPLC analysis found that there were a large number of side products generated (Figure 5(a)), some of which were likely to be oligomeric species caused by undesired cross-linking within the solid support. Nevertheless, the last ten residues were coupled under microwave-assisted conditions, followed by cleavage and deprotection. As expected, the crude material consisted of multiple products and a two-step purification was again employed as described above in order to isolate the semipure reduced species. Treatment with two equivalents of iodine gave the desired product, and a second two-step purification was performed, due to the complexity of the mixture. Despite the low yield, sufficient purified peptide was isolated (Figures 5(b) and 5(c)) for evaluation in the binding assay.

Both the ΔA25/26 human INSL3 monolactam A24-B22 and bis-lactam A11-B10/A24-B22 analogues were tested in the RXFP2 competition binding assay (Table 1). The receptor binding affinity of each was found to be 8.35 ± 0.11 and 7.92 ± 0.12, respectively. While these values were approximately 10-fold and 12-fold lower compared to native INSL3 (9.24 ± 0.02), the more relevant comparison is with ΔA25,26 human INSL3 (8.59 ± 0.06) which shows that both analogues are only 1.7-fold and 4.6-fold lower. This showed that employing lactam bridges as stable cystine isosteres in insulin-like peptides holds much promise. The very modest reduction in binding affinity of ΔA25/26 human INSL3 monolactam A24-B22 compared to ΔA25/26 human INSL3 is consistent with similar observations recently made for synthetic human INSL3 monolactam A24-B22 versus native INSL3 [29]. The reason for this is probably due to a subtle change in the conformation that is caused by one or more of the following: the shorter length of the amide bond compared to the disulfide bond, change in the electronic nature of the cystine mimic, and steric effects. Unfortunately, insufficient material was obtained to perform structural studies using CD or NMR spectroscopy [38, 39]. It was also not possible to perform cAMP activity-based assays or, equally important, plasma stability assays in order to verify whether the more stable amide bond(s) gives the model peptides enhanced stability. Yet importantly, this study demonstrates the feasibility of acquiring such heterodimeric analogues although further work is clearly required to improve their total synthesis and subsequent yields of these analogues. This will include employing different protecting group combinations (e.g., Allyl/Alloc), incorporating a solution-phase step, or using a very low loading resin to minimize both steric crowding during assembly and the nonspecificity of the on-resin directed lactam bond formation [40].

In conclusion, a novel “two-chain assembly” strategy on a solid support was developed for synthesising complex bis-lactam interchain-linked heterodimeric insulin-like peptides for the first time. This was achieved by use of microwave-assisted SPPS and use of semiorthogonal side-chain protecting groups. This approach enabled the preparation of both mono- and bis-lactam analogues of ΔA25/26 human INSL3 which were subsequently shown to have significant binding affinity at nanomolar concentration for RXFP2 receptor. 

## Figures and Tables

**Table 1:** Receptor binding affinities (pK_*i*_) of INSL3 and analogues. Data for entry 2 taken from reference [32].

Peptide	Receptor binding pK_*i*_ (*n*=)
Human INSL3	9.24 ± 0.02 (6)
ΔA25/26 human INSL3	8.59 ± 0.06 (3)
ΔA25/26 human INSL3 mono-lactam A24-B22	8.35 ± 0.11 (3)
ΔA25/26 human INSL3 bis-lactam A11-B10, A24-B22	7.92 ± 0.12 (3)

## Abbreviations

Acm: Acetamidomethyl
Alloc: Allyloxycarbonyl
Boc: *tert*-butyloxycarbonyl
Bzl: Benzyl
CD: Circular dichroism
Dab: 2,4-Diaminobutyric acid
DCM: Dichloromethane
Dde: (4,4-Dimethyl-2,6-dioxocyclohex-1-ylidene)ethyl
DIC: Diisopropylcarbodiimide
DIPEA: Diisopropylethylamine
DMF: Dimethylformamide
DODT: 3,6-Dioxa-1,8-octane-dithiol
Dpr: 2,3-Diaminopropionic acid
ESI-MS: Electrospray ionization mass spectrometry
Fmoc: 9-Fluorenylmethoxycarbonyl
HATU: 1-[Bis (dimethylamino) methylene]-1*H*-1,2,3-triazolo [4,5-b] pyridinium 3-oxid hexafluorophosphate
HEK: Human embryonic kidney
HOBt: 1-Hydroxybenzotriazole
INSL3: Insulin-like peptide 3
ivDde: 1-(4,4-Dimethyl-2,6-dioxocyclohex-1-ylidene)-3-methylbutyl
Mmt: p-Methoxytrityl
Mtt: 4-Methyltrityl
NMP: 1-Methyl-2-pyrrolidinone
NMR: Nuclear magnetic resonance
2-PhiPr: 2-Phenylisopropyl
Dmab: {N-[1-(4,4-Dimethyl-2,6-dioxocyclohexylidene)-3-methylbutyl]-amino
Fm: 9-Fluorenylmethyl
PEG-PS: Polyethylene glycol-polystyrene
RP-HPLC: Reversed-phase high performance liquid chromatography
RXFP2: Relaxin family peptide receptor 2
SPPS: Solid-phase peptide synthesis
tBu: *tert*-butyl
TFA: Trifluoroacetic acid
TIPS: Triisopropylsilane
Trt: Trityl.
